# Relationship between Longitudinal Upper Body Rotation and Energy Cost of Running in Junior Elite Long-Distance Runners

**DOI:** 10.3390/sports11100204

**Published:** 2023-10-18

**Authors:** Charlotte Lang, Axel Schleichardt, Frank Warschun, Nico Walter, Daniel Fleckenstein, Fides Berkel, Olaf Ueberschär

**Affiliations:** 1 Institute for Biomechanics, ETH Zürich, 8092 Zurich, Switzerland; charlotte.lang@hest.ethz.ch; 2 Institute for Applied Training Science, 04229 Leipzig, Germany; schleichardt@iat.uni-leipzig.de (A.S.); warschun@iat.uni-leipzig.de (F.W.); walter@iat.uni-leipzig.de (N.W.); fleckenstein@iat.uni-leipzig.de (D.F.); berkel@iat.uni-leipzig.de (F.B.); 3 Department of Engineering and Industrial Design, Magdeburg Stendal University of Applied Sciences, 39114 Magdeburg, Germany

**Keywords:** running, energy expenditure, running efficiency, upper trunk, lower trunk, movement, inertial measurement units, biomechanics, elite

## Abstract

Running is a basic form of human locomotion and one of the most popular sports worldwide. While the leg biomechanics of running have been studied extensively, few studies have focused on upper-body movement. However, an effective arm swing and longitudinal rotation of the shoulders play an important role in running efficiency as they must compensate for the longitudinal torques generated by the legs. The aim of this study is to assess the upper-body rotation using wearable inertial sensors and to elucidate its relation to energy expenditure. Eighty-six junior elite middle- and long-distance runners (37 female, 49 male) performed an incremental treadmill test with sensors attached on both shoulders, tibiae and the sacrum. The mean and total horizontal shoulder and pelvis rotations per stride were derived while energy costs were determined using respiratory gas analysis and blood sampling. Results show that shoulder and pelvis rotations increase with running speed. While shoulder rotation is more pronounced in female than in male runners, there is no sex difference for pelvis rotation. The energy cost of running and upper trunk rotation prove to be slightly negatively correlated. In conclusion, upper body rotation appears to be an individual characteristic influenced by a sex-specific body mass distribution.

## 1. Introduction

Bipedal walking and running are the natural forms of ground locomotion for the human species. They have been learned and motorically mastered with ease since the early stages of childhood and remain an integral part of everyday life for virtually all healthy human beings throughout their lifespan. In particular, running belongs to the most popular recreational and amateur sports in many developed countries as it combines easy access for the public and offers efficient physical and mental benefits [1,2]. Moreover, running is part of the Olympic programme in numerous disciplines, and every year thousands of competitive runners take part in city races around the world.

Despite its popularity, running is associated with an unfavourably high injury prevalence, especially with respect to overuse injuries of the lower limbs. According to the literature, from 27% to 70% of runners experience at least one such injury per year, while the incidence reaches values of up to 33 injuries per 1000 h of running [1,3,4,5,6]. The lower limbs are by far the most common localisations of running-related injuries, with their share exceeding 94% [7]. In general terms, this high risk of injury is inherently linked to continuous high-impact and active forces during running, even though the underlying individual biomechanical mechanisms may be complex and no simple direct causal relationships could be identified [7]. Given these epidemiological figures, it is understandable that much of the recent research has focused on leg kinematics and kinetics [5,8,9,10,11] rather than on whole-body movement. In addition, it is well documented that lower vertical oscillation and higher leg stiffness are associated with better running efficiency [11,12,13], which is an important determinant of running performance [14]. While these kinematic parameters are mainly related to leg biomechanics, there is also evidence for the important role of upper body movement [15,16]. However, only a few studies have explicitly addressed the upper body biomechanics of running in detail. In essence, a holistic biomechanical picture of running is worth considering in order to optimise performance in elite sports [17,18], to prevent injuries [1,3,5] and even to improve concepts of bipedal locomotion of humanoid robots in engineering [19,20].

The upper body movement that occurs during running is dominated by the arm swing, which is (often subconsciously) adapted in timing and amplitude to the leg swing to compensate for the torques around the longitudinal axis of the body generated by the legs. The arm swing, together with the longitudinal rotation of the shoulders (in relation to the pelvis), must therefore be performed actively in order to maintain balance efficiently. It is worth noting that the additional metabolic cost of this is less than the energy loss of the whole-body movement would be without an active arm swing. Thus, the total energy expenditure is reduced by an active arm swing [21]. Convincing experimental evidence was provided by Arellano and Kram, who showed that a proper arm swing substantially reduces the total energy cost as well as the peak-to-peak shoulder amplitude and upper trunk rotation [22,23]. In contrast, excessive arm movement and trunk rotation may have a negative effect on energy expenditure and thus on running efficiency [11].

Although coaches in junior elite running are usually aware of this basic biomechanical background, they often feel unable to judge whether a particular form of individual arm swing is efficient or should be optimised. In the absence of evidence-based data as a reference for “individually appropriate” amplitudes of longitudinal trunk rotation, the coaches thus must rely on schematic expert knowledge based on a visual inspection and experience, which admittedly lacks specificity. It is therefore a promising approach to individually objectify longitudinal trunk rotation (“trunk twist”) to assess arm swing and overall running economy for improving running technique and economy in a competitive context. As a change in the movement pattern is generally easier to adapt to in younger training ages, this optimisation process may produce the best results in junior elite runners rather than in runners at their career peaks.

This study addresses the idea of a quantitative assessment of upper body rotation in running and tries to bridge the existing knowledge gap between biomechanical theory and exercise practice with respect to efficiency in competitive junior elite long-distance runners. The first purpose of this study is thus to obtain statistical reference values for the longitudinal rotation of the upper trunk (in terms of shoulder motion) and the lower trunk (in terms of sacrum motion) for junior elite middle- and long-distance runners and to analyse the effects of sex and speed. Second, by measuring the energy cost of running (*C*_r_) via oxygen consumption and lactate production for the same cohort under laboratory conditions, this study investigates the relation between trunk twist and *C*_r_, addressing the question of whether there is a generally optimal configuration. Altogether, this study intends to further promote the in-field use of wearables for assessing movement patterns in running to support competitive athletes in training and in tapping their full individual potential, following the general concepts pointed out by Camomilla et al. [24].

## 2. Materials and Methods

### 2.1. Subjects

Eighty-six junior elite middle- and long-distance runners (37 females: 16.8 ± 0.3 years, 169.4 ± 5.5 cm, 52.2 ± 5.3 kg, BMI 18.2 ± 1.7 kg m^−2^; 49 males: 19.2 ± 0.2 years, 181 ± 5.6 cm, 67 ± 6.5 kg, BMI 20.3 ± 1.4 kg m^−2^) participated in this study. Maximum oxygen uptake and maximum heart rate were determined for 56 out of the 86 athletes in a test to volitional exhaustion, yielding 58.2 ± 3.9 mL kg^−1^ min^−1^ for the females and 66.8 ± 4.2 mL kg^−1^ min^−1^ for the males, confirming their trained physical condition [25]. All athletes were part of the German junior national team at the time of measurements. Detailed data on the subjects can be found in the Table A1, Table A2 and Table A3 in the Appendix A.

The study was conducted according to the guidelines of the Declaration of Helsinki and approved by the ethics commission of the Department of Engineering and Industrial Design at the Magdeburg-Stendal University of Applied Sciences under reference number EKIWID-2023-04-001SA. Written informed consent was obtained from all participants.

### 2.2. Test Design and Protocol

All subjects completed an incremental treadmill test at the Institute for Applied Training Science (IAT) in Leipzig, Germany, as part of their regular performance diagnostics programme. The subjects were instructed to avoid intensive training loads two days before the test and to perform only easy warm-up exercises. The majority of the subjects were familiarized with the test. If an athlete was taking part in the test for the first time, a familiarisation run of 2 min was performed before the beginning of the incremental treadmill test. The last meal was within 3 h before starting the test. The temperature in the laboratory was controlled (18–20 °C).

The treadmill test consisted of four stages of either 4 times 2000 m (for middle-distance runners) or 4 times 3000 m (for long-distance runners) with a rest of one minute in between. After each break, the speed was increased by 0.25 m s^−1^. The starting speed was individually set according to the subject’s performance level. For experienced runners, that starting speed was chosen such that the 3 mmol lactate threshold was exceeded at the third stage according to previous treadmill tests on the same subject [26]. For runners participating in this diagnostic setting for the first time, the starting speed was approximated by the speed level of comparable athletes based on the expert assessment by their coaches. Out of the 86 athletes, 86 completed stages 1 and 2, 84 completed stage 3 and 82 athletes completed stage 4. Regarding all runners that completed an additional test to volitional exhaustion, the mean oxygen uptakes and mean heart rates in the incremental treadmill test confirmed submaximal loading, with 73.1 ± 5.3% of maximum oxygen uptake and 85.3 ± 3.1% of maximum heart rate at stage 1 to 88.4 ± 7.1% and 96.4 ± 2.9% at stage 4.

### 2.3. Data Acquisition and Pre-Processing

For each participant, triaxial magneto-inertial measurement units (MTw Awinda, Xsens Technologies BV, Enschede, Netherlands) were fixed non-invasively at the distal anteromedial sections of the right and left tibiae (2 sensors), on the centre of each shoulder’s spina scapula (2 sensors) and at the upper S1 section of the sacrum using Leukotape^®^ and Velcro^®^ straps. Three-dimensional accelerations, rotational speeds and Euler angles of sensor orientation with respect to the laboratory frame of reference (global frame) were acquired with 1.000 Hz and downsampled by sensor fusion to 120 Hz. Pelvis and shoulder yaw angles, along with their three-dimensional total changes in Euler angles, served as measures for pelvis and shoulder total rotation in 1D and 3D during each running step:

First, *mean horizontal shoulder rotation per stride* (HSR in degrees) is defined as the angular range of the cyclic oscillation of the scapular (scap) sensor’s yaw angle $\varphi_{\mathrm{scap}}\left(t\right)$ around the global longitudinal axis (which corresponds to a good approximation to the runner’s longitudinal axis):

(1) $$\mathrm{HSR}:={\langle \underset{\tau \in \left[t_{i-1},t_{i}\right]\;}{\max}\left(\varphi_{\mathrm{scap}}\left(\tau \right)\right)-\underset{\tau \in \left[t_{i-1},t_{i}\right]\;}{\min}\left(\varphi_{\mathrm{scap}}\left(\tau \right)\right)\rangle }_{i}$$

Here, i=1, 2, 3, … denotes the consecutive stride index and $\left[t_{i-1},t_{i}\right]$ its time interval in seconds. The mean ${\langle \ldots \rangle }_{i}\equiv \frac{1}{n}\sum_{i=1}^{n}\ldots$ is evaluated over all strides of the bout.

Second, given the three degrees of rotational freedom of the glenohumeral joint, the analysis of shoulder rotation can be extended to 3D, i.e., combining all three spatial axes. For this purpose, the *mean total shoulder rotation per stride* (TSR in degrees) is computed as the mean absolute sum of angular rotations with global-frame rotational speeds $\vec{\omega }={\left(\omega_{x},\omega_{y},\omega_{z}\right)}^{T}$$={\left({\dot{\varphi }}_{\mathrm{scap},x},{\dot{\varphi }}_{\mathrm{scap},y},{\dot{\varphi }}_{\mathrm{scap},z}\right)}^{T}$ of the shoulder sensors around their axes:

(2) $$\mathrm{TSR}:={\langle \int_{\tau \in \left[t_{i-1},t_{i}\right]}^{}\left(\left|\omega_{x}\left(\tau \right)\right|+\left|\omega_{y}\left(\tau \right)\right|+\left|\omega_{z}\left(\tau \right)\right|\right)d\tau \rangle }_{i}$$

Third, *mean horizontal pelvis rotation per stride* (HPR in degrees) is computed in analogy to HSR and Equation (1) based on the cyclic longitudinal oscillation of the sacrum (sac) sensor’s yaw angle. Fourth, *mean total pelvis rotation per stride* (TPR in degrees) corresponds to the TSR in terms of the rotational speed of the sacrum sensor ${\left({\dot{\varphi }}_{\mathrm{sac},x},{\dot{\varphi }}_{\mathrm{sac},y},{\dot{\varphi }}_{\mathrm{sac},z}\right)}^{T}$ in analogy to Equation (2).

To analyse metabolic energy consumption, oxygen uptake rates (${\dot{V}}_{O_{2}}$) and carbon dioxide output rates (${\dot{V}}_{{\mathrm{CO}}_{2}}$) were measured breath-by-breath during the last 60 s of each stage using a stationary respiratory gas analysis system (MetaMax 3B, Cortex Biophysik GmbH, Leipzig, Germany). The highest 30 s average was used for further analysis. Capillary blood samples of 20 µL were taken from the ear lobe after each stage, solubilised in a 1000 μL hemolysate solution and analysed for their lactate level (La) using a stationary system (SUPER GL, Dr. Müller Gerätebau GmbH, Freital, Germany). Specific aerobic and anaerobic lactic energy expenditure (J kg^−1^) and distance-normalised *C*_r_ (J kg^−1^ m^−1^) were calculated using ${\dot{V}}_{O_{2}}$, respiratory exchange rate $RER={\dot{V}}_{{\mathrm{CO}}_{2}}/{\dot{V}}_{O_{2}}$ and La following standard procedures [27,28,29]. To enable comparisons with data from the pertinent literature, gross energy expenditure was analysed, including both resting and working metabolic rates.

### 2.4. Data Analysis

Statistical analyses were conducted in SPSS [30]. Because Levene tests for homogeneity of variances and Kolmogorov–Smirnov tests for normality yielded negative results (*p* < 0.05) for the majority of the considered parameters when separated by sex, non-parametric Friedman tests for repeated measures were employed to detect differences between speed stages within each sex group, followed by Dunn–Bonferroni post hoc procedures for speed stages. As for differences between sexes the Mann–Whitney U test was applied at each speed stage. Effect sizes *r* were calculated using the test statistic *z* [31] and interpreted following Cohen [32], with 0.1 < *r* < 0.3 representing weak, 0.3 < *r* < 0.5 moderate and *r* ≥ 0.5 strong effects. Correlation analyses were conducted in R [33]. Spearman’s partial correlation coefficient *r*_S_ was used to evaluate the relation between individual trunk twist and running economy. For this, the speed stage (e.g., 1, 2, …) rather than the nominal running speed (e.g., 3.00 m s^−1^) was set as the controlled variable to account for the differing starting speeds of the athletes in the first stage and to normalise the influence of prior stages (for stages 2, 3 etc.). As above, the interpretation of *r**_S_*** follows the suggestions of Cohen [32], with weak, moderate and strong correlations for 0.1 ≤ *r**_S_*** < 0.3, 0.3 ≤ *r**_S_** <* 0.5 and *r**_S_*** ≥ 0.5, respectively. The level of significance was set to *p* < 0.05.

## 3. Results

Please note that detailed measurement results for all studied aspects of the running dynamics and energetics of the cohort are provided in Table A4, Table A5, Table A6, Table A7, Table A8, Table A9, Table A10, Table A11, Table A12, Table A13 and Table A14 in Appendix A for reference. This includes step frequency, ground contact time, time of flight, amplitude of vertical oscillations, elevation of sacrum during flight, step length, oxygen uptake, blood lactate accumulation, aerobic, anaerobic and total contributions to *C*_r_.

### 3.1. Shoulder Rotation

**Figure 1:**
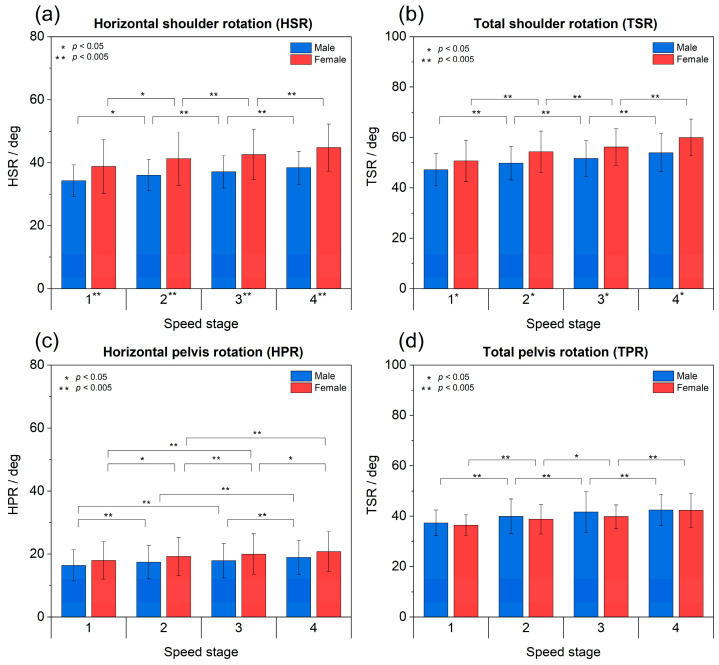
Mean rotation angles per stride as a function of running speed (stages 1 to 4) and sex: (**a**) HSR (1D), (**b**) TSR (3D), (**c**) HPR (1D), (**d**) TPR (3D). Error bars represent ±1 standard deviation while stars (*, **) denote levels of significance. Stars at speed stage numbers denote levels of significance for differences between sexes.

The amplitudes of mean HSR increase with running speed (Figure 1a, Table 1; *p* < 0.001) and are generally higher for females than for males at each speed stage (*p* < 0.001). HSR values range from 38.8° at stage 1 to 44.8° at stage 4 (+6.0° ≙ +15%) for females and from 34.3° to 38.4° (+4.1° ≙ +12%) for males. Post hoc tests confirm moderate increases in HSR between almost all stages for both sexes (stages 2–4: *p* < 0.025; 0.11 ≤ *r* ≤ 0.49, the only exception being stage 1 vs. 2). Similarly, mean TSR increases with running speed (Figure 1b, Table 2; *p* < 0.001) and exhibits higher values for females as compared to males (*p* < 0.01). TSR values range from 50.7° to 59.9° (+9.3° ≙ +18% increase from stage 1 to 4) for females and from 47.2° to 53.9° (+6.8° ≙ +14%) for males. Post hoc tests confirm significant differences in TSR between the speed stages with medium to strong effect sizes (*p* < 0.025; 0.14 ≤ *r* ≤ 0.52).

In summary, female runners exhibit higher values of shoulder rotation (average difference of +12%) and higher absolute increases of shoulder rotation with running speed (+18% vs. +14%) than their male counterparts for an increase in speed from stage 1 to 4 of roughly +26%.

### 3.2. Pelvis Rotation

The mean HPR per stride (Figure 1c, Table 3) increases with the running speed for both sexes (Figure 1c; *p* < 0.001), growing from 17.9° at stage 1 to 20.8° at stage 4 (+2.9° ≙ +16%) for females and from 16.4° to 18.9° (+2.5° ≙ +15%) for males. Interestingly, no significant differences between sexes are observed here. Post hoc tests confirm between-stage increases with small to medium effect sizes (females: *p* < 0.025; 0.16 ≤ *r* ≤ 0.40; males: *p* < 0.05; 0.11 ≤ *r* ≤ 0.30) and only a few non-significant.

Similarly, the mean TPR per stride grows with running speed (Figure 1d, Table 4; *p* < 0.001) with no significant differences between sexes. The TPR ranges from 36.5° at stage 1 to 42.3° at stage 4 (+5.8° ≙ +16%) for females and from 37.3° to 42.5° (+5.2° ≙ +13%) for males. Post hoc tests confirm significant differences among all speed stages with small to moderate to strong effect sizes (females: *p* < 0.05; 0.15 ≤ *r* ≤ 0.51; males: *p* < 0.01; 0.13 ≤ *r* ≤ 0.42). Hence, pelvis rotation is comparable between sexes and increases by approximately 15% for an increase of speed of +26%.

### 3.3. Energy Cost of Running

**Figure 2:**
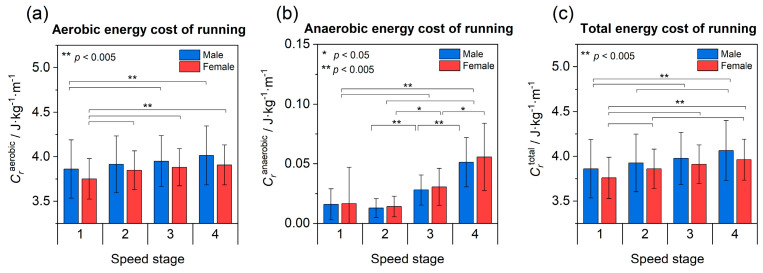
Aerobic (**a**) and anaerobic (**b**) contributions along with total *C*_r_ (**c**) as functions of running speed (stages 1−4) and sex (colour). Error bars depict one standard deviation. Please note the altered scaling of panel (**b**).

As expected, the total energy expenditure $C_{r}^{\mathrm{total}}$ increases with running speed, from 3.76 J kg^−1^ m^−1^ at stage 1 to 3.96 J kg^−1^ m^−1^ at stage 4 for females (+0.2 J kg^−1^ m^−1^ ≙ +5%) and from 3.86 J kg^−1^ m^−1^ to 4.06 J kg^−1^ m^−1^ (+0.2 J kg^−1^ m^−1^ ≙ +5%) for males (Figure 2a–c; *p* < 0.005). Post hoc tests confirm small to medium changes between stages (females: *p* ≤ 0.005; 0.17 ≤ *r* ≤ 0.36; males: *p* < 0.001; 0.19 ≤ *r* ≤ 0.29). Although the mean total $C_{r}^{\mathrm{total}}=C_{r}^{\mathrm{aerob}}+C_{r}^{\mathrm{anaerob}}$ appears to visually differ between sexes, there are no significant differences between the sexes, neither in $C_{r}^{\mathrm{total}}$ nor in its aerobic or anaerobic contributions $C_{r}^{\mathrm{aerob}}$ or $C_{r}^{\mathrm{anaerob}}$, respectively. Furthermore, the relative change of +5% for an increase in speed of about +26% is the same for females and males.

Studying the dominant aerobic contribution to *C*_r_ (Figure 2a) shows that running speed remains a significant factor for both sexes (*p* < 0.005), growing from 3.75 to 3.91 J kg^−1^ m^−1^ for females (*p* ≤ 0.005) with small- to medium-sized effects (0.20 ≤ *r* ≤ 0.33) between stages. For males, aerobic energy expenditure rises from 3.84 to 4.02 J kg^−1^ m^−1^ (*p* < 0.001) with smaller or no significant effects between stages (0.20 ≤ *r* ≤ 0.21).

As regards the anaerobic contribution to energy expenditure (Figure 2b), the female runners exhibit significant increases with medium to strong effect sizes between most stages (*p* ≤ 0.05; 0.23 ≤ *r* ≤ 0.52; only exception from stage 1 to 2), ranging from 0.017 at stage 1 to 0.056 J kg^−1^ m^−1^ at stage 4. Similarly, small to moderate effects are observed for males (*p* ≤ 0.001; 0.18 ≤ *r* ≤ 0.41) within a range from 0.016 at stage 1 to 0.051 J kg^−1^ m^−1^ at stage 4.

### 3.4. Relations between Trunk Rotation and Running Economy

**Figure 3:**
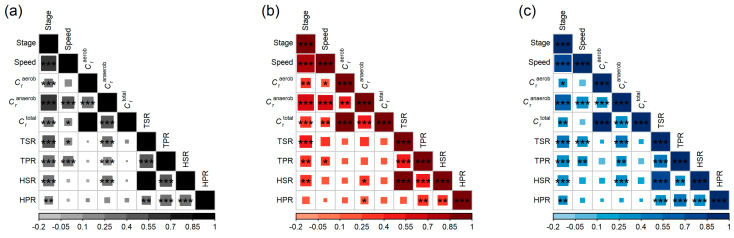
Relations between all relevant study variables in terms of Spearman’s correlation coefficients, depicted in a heat map for (**a**) all, (**b**) female and (**c**) male runners, together with their significances (* denoting *p* < 0.05, ** *p* < 0.01 and *** *p* < 0.001).

All key variables of this study (running speed, HSR, TSR, HPR, TPR, contributions to *C*_r_) were analysed for interrelations using Spearman’s correlation coefficient (Figure 3), both separated by sex and in the combination of both sexes. In summary, all parameters show positive medium to strong correlations with the speed stage (females: 0.12 ≤ *r*_S_ ≤ 0.64; males: 0.17 ≤ *r*_S_ ≤ 0.60). Furthermore, there are small positive correlations between HSR and HPR (females: *r*_S_ = 0.24, *p* < 0.01; males: *r*_S_ = 0.29, *p* < 0.001) and medium positive correlations between TSR and TPR (females: *r*_S_ = 0.44, *p* < 0.001; males: *r*_S_ = 0.40, *p* < 0.001), supporting the introduced hypothesis of a “twisting trunk”.

Considering running economy, HSR seems to be linked to *C*_r_ in terms of positive but small correlations to the anaerobic fraction of energy expenditure for both female (0.08 ≤ *r*_S_ ≤ 0.19) and male runners (0.02 ≤ *r*_S_ ≤ 0.29) and for both sexes combined.

**Figure 4:**
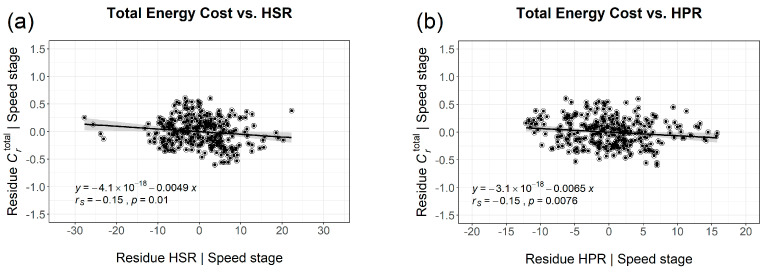
Confounder−corrected, true relation between total $C_{r}^{\mathrm{total}}$ and trunk rotation ((**a**) HSR, (**b**) HPR)) for the combined cohort in terms of residues after regression analysis with speed stage.

**Figure 5:**
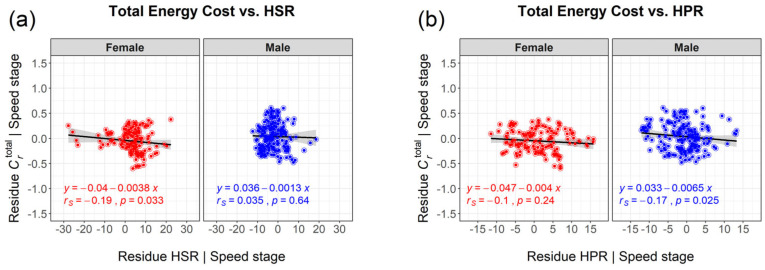
Confounder−corrected, true relation between total $C_{r}^{\mathrm{total}}$ and trunk rotation ((**a**) HSR, (**b**) HPR), separated by sex and depicted as residues after regression analysis with speed stage.

However, because all key variables of this study are significantly influenced by running speed (in terms of speed stage), spurious correlations are likely to occur, especially with respect to the impact of trunk rotation on energy expenditure, where effect sizes are intrinsically small. Therefore, the effects of HSR, TSR, HPR and TPR on *C*_r_ were analysed by partial correlation analysis, eliminating the influence of the confounder speed stage by employing regression analysis. Results of this elimination are shown in Figure 4 for all runners in a pooled cohort and in Figure 5 where they are separated by sex.

As depicted in Figure 4, there is, in contrast to the spurious positive correlation at first glance (Figure 3), a small negative correlation between horizontal trunk rotation in terms of HSR, HPR and total energy costs of running (*r*_S_ = −0.15; *p* < 0.01 for HSR and *p* < 0.008 for HPR) in the pooled cohort. When separated by sex, only the negative correlation between shoulder rotation (HSR) and the total energy expenditure is significant (*r*_S_ = −0.19; *p* = 0.033) for females (Figure 5). In contrast, for males only pelvis rotation (HPR) is negatively correlated to $C_{r}^{\mathrm{total}}$ (*r*_S_ = −0.17; *p* < 0.025). In summary, the negative correlation coefficients imply, despite being small, that the energy cost of running *decreases* with more pronounced horizontal rotation of the trunk (i.e., HSR for females and HPR for males).

## 4. Discussion

This study has produced three main findings on upper (scapula) and lower (pelvis) trunk rotation in terms of longitudinal and total angles, sex, running speed and running economy.

The first finding, which relates directly to the amplitude of upper trunk rotation, is that there is a highly significant sex difference in both HSR and TSR. Higher shoulder rotation amplitudes are observed for female runners, while amplitudes increase with speed for both sexes (+18% vs. +14% for female vs. male runners). The second finding is that the rotation of the lower trunk in terms of horizontal and TPR shows *no* relevant differences between the sexes, while it increases with running speed to a similar extent (+15%) as shoulder rotation. This intriguing sex difference may be due to anthropometric reasons: The average male runner’s trunk has a higher mass and a greater proportion of muscle mass compared to females, whereas the female trunk is generally lighter but has a higher proportion of fat mass, mainly because of their breasts [34]. As a result, the female runners’ trunk possesses a lower longitudinal moment of inertia and thus experiences higher rotational accelerations for a given external leg torque. Moreover, female runners’ have a smaller shoulder span, resulting in shorter lever arms, so higher momenta of the upper limbs need to be generated to achieve the same upper-body counter torque. Female runners therefore need to compensate for this anthropometric condition by using a faster and more intense arm swing with higher shoulder rotation amplitudes. This effect may be further amplified by the greater proportion of wobbling masses in their upper trunk due to their body composition. In contrast, a heavier, stiffer trunk in males with a wider shoulder span provides more dynamic stability in balancing rotational movements between the upper and lower extremities. In addition, a generally greater hip flexibility of female runners, particularly in terms of the hip extension at toe-off [11,15,35], may cause higher leg torques and thus further contribute to more intense upper-body balancing rotations.

The third finding of this study is the small but surprisingly negative true correlation between trunk rotation and *C*_r_. Counterintuitively, a higher degree of shoulder rotation does *not* imply a higher *C*_r_ but rather a smaller *C*_r_. Moreover, following Hinrichs et al. [21], the arms contribute only by 5–10% to the vertical oscillations of the whole body, with vertical oscillations being one of the two major contributors to energy expenditure during running. Considering this, the inter-individual differences in shoulder rotation amplitudes observed in our cohort are too small to explain the substantial variance in their running economy. Hence, the evidence suggests that the trained cohort examined in this study has well-balanced, individually optimised rotational movement patterns of their trunks, such that the degree of trunk rotation does not have an apparent pervasive effect on running economy. These findings are supported by those of Anderson [36], who mentions the importance of a faster shoulder rotation for better running economy. In essence, we suggest that the “optimal” longitudinal trunk rotation varies individually and widely by following the common aim of balancing longitudinal torques between individually proportioned lower and upper body inertia. The substantial correlation between total upper and lower trunk motion found for this cohort supports this hypothesis (see Figure 3).

From a technical point of view, the outcome of this study supports the concept of using IMU sensors as a promising tool for investigating segmental motion during running beyond the lower limbs. In the future, IMUs might potentially be used to elucidate energy costs and running efficiency more accurately based on highly time-resolved acceleration data for all relevant body segments involved. As a first step, follow-up studies could include additional segments, especially the lower and upper arms as well as the head for IMU-based running gait analysis. Further parameters such as vertical oscillations, pelvic roll angle as measures of “pelvic instability” and hip extension at toe-off should also be considered. Finally, as the extrapolation of our findings from junior elite to other levels of performance may be difficult to justify, it would be of interest to investigate other relevant populations, e.g., amateur runners. In such less trained groups, the degree of trunk rotation may be differently related to energy expenditure.

For the time being, however, our results confirm that any blanket assessment of the quality of trunk rotation during running, such as “the less, the better”, is unwarranted and should be avoided from a biomechanical point of view, especially in junior elite sport, where there must always be room for individual optimisation through training experience.

The results of the current study are limited by the small sample size, especially with regard to the differences in starting speed. The test was performed as a part of a regular performance diagnostic programme. Due to this demanding test setting, the results may have been influenced by the onset of fatigue during the incremental treadmill test. Moreover, this study focuses on upper body biomechanics only. Future studies should attempt to examine whole-body movement in a holistic approach, which would obviously require additional IMU sensors and extensive analysis of joint angles and further angular velocities.

## 5. Conclusions

This study contributes to the understanding of upper body movement during running and its relationship to energy expenditure. Our results show that trunk rotation is speed-dependent and increases significantly with the progression of running speed. However, the amount of shoulder and pelvis rotation appears to be highly individual and, as suggested by the results of this study, is strongly influenced by sex-specific mass ratios between upper and lower body segments. Larger angles of rotation do not necessarily imply increased energy expenditure, contrary to what might be intuitively expected. As shown for the investigated cohort of elite junior runners with strong training and competition expertise, *high* rotational amplitudes may *reduce* the required energy costs of running and thereby *elevate* running efficiency. We, therefore, recommended that young runners should not be generally restricted in their upper body range of motion by their coaches but should instead be allowed to find their individual optimum through training experience with regard to effective support of the leg biomechanics and a reduced energy cost of running.

## Figures and Tables

**Table 1:** Mean HSR per stride (mean± SD, degrees); athletes subdivided according to starting speed.

Performance Group (Starting Speed)	Sex		Speed Stage			
			1	2	3	4
1 (3.00/3.25 m/s)	male	-	-	-	-	-
	female	*n* = 13	37.4 ± 12	41.0 ± 12.2	41.7 ± 11.1	42.3 ± 10.5
2 (3.50/3.75 m/s)	male	*n* = 11	32.3 ± 3.9	34.4 ± 4.7	35.5 ± 5.0	36.2 ± 3.6
	female	*n* = 21	40.3 ± 5.0	41.9 ± 4.9	43.7 ± 4.9	45.8 ± 5.2
3 (4.00/4.25 m/s)	male	*n* = 31	34.9 ± 5.3	36.6 ± 5.1	37.7 ± 5.3	39.1 ± 5.6
	female	*n* = 2	32.3 ± 12.1	35.5 ± 14.3	37.1 ± 15.1	52.3 *
4 (4.50/4.75 m/s)	male	*n* = 5	34.4 ± 5.5	36.1 ± 5.0	37.3 ± 5.7	38.4 ± 5.1
	female	-	-	-	-	-

* *n* = 1.

**Table 2:** Mean TSR per stride (mean ± SD, degrees); athletes subdivided according to their starting speed.

Performance Group (Starting Speed)	Sex		Speed Stage			
			1	2	3	4
1 (3.00/3.25 m/s)	male	-	-	-	-	-
	female	*n* = 13	49.7 ± 11.0	54.0 ± 11.3	55.3 ± 9.5	58.1 ± 10.2
2 (3.50/3.75 m/s)	male	*n* = 11	43.3 ± 4.6	45.9 ± 5.5	48.7 ± 6.0	51.5 ± 6.1
	female	*n* = 21	51.9 ± 5.6	55.2 ± 5.7	57.4 ± 5.2	60.7 ± 5.5
3 (4.00/4.25 m/s)	male	*n* = 31	47.6 ± 6.3	50.3 ± 6.6	51.8 ± 7.1	54.1 ± 7.8
	female	*n* = 2	43.7 ± 9.5	47.4 ± 11.3	50.0 ± 11.6	63.5 *
4 (4.50/4.75 m/s)	male	*n* = 5	52.9 ± 5.6	55.3 ± 5.7	57.1 ± 6.2	58.7 ± 7.0
	female	-	-	-	-	-

* *n* = 1.

**Table 3:** Mean HPR per stride (mean ± SD, degrees); athletes subdivided according to starting speed.

Performance Group (Starting Speed)	Sex		Speed Stage			
			1	2	3	4
1 (3.00/3.25 m/s)	male	-	-	-	-	-
	female	*n* = 13	18.4 ± 7.7	20.0 ± 7.8	20.9 ± 8.3	21.6 ± 8.1
2 (3.50/3.75 m/s)	male	*n* = 11	16.6 ± 5.0	17.4 ± 5.3	18.0 ± 5.0	19.5 ± 3.3
	female	*n* = 21	17.7 ± 5.1	18.7 ± 5.0	19.5 ± 5.5	20.1 ± 5.4
3 (4.00/4.25 m/s)	male	*n* = 31	16.3 ± 5.3	17.3 ± 5.6	17.8 ± 5.9	18.6 ± 6.1
	female	*n* = 2	17.5 ± 4.9	18.9 ± 5.4	19.6 ± 5.3	24.0 *
4 (4.50/4.75 m/s)	male	*n* = 5	16.4 ± 3.8	17.5 ± 4.2	18.0 ± 3.7	19.1 ± 3.0
	female	-	-	-	-	-

* *n* = 1.

**Table 4:** Mean TPR per stride (mean ± SD, degrees); athletes subdivided according to starting speed.

Performance Group (Starting Speed)	Sex		Speed Stage			
			1	2	3	4
1 (3.00/3.25 m/s)	male	-	-	-	-	-
	female	*n* = 13	36.9 ± 4.3	39.0 ± 5.0	40.2 ± 5.3	42.2 ± 6.1
2 (3.50/3.75 m/s)	male	*n* = 11	34.9 ± 4.4	36.7 ± 4.3	38.7 ± 4.5	40.3 ± 4.5
	female	*n* = 21	36.3 ± 4.0	38.8 ± 6.6	39.8 ± 4.5	42.2 ± 7.3
3 (4.00/4.25 m/s)	male	*n* = 31	37.6 ± 4.9	40.4 ± 7.0	42.0 ± 8.6	42.6 ± 6.2
	female	*n* = 2	34.6 ± 4.2	37.1 ± 5.3	38.7 ± 5.3	44.0 *
4 (4.50/4.75 m/s)	male	*n* = 5	40.7 ± 6.1	43.6 ± 9.2	45.5 ± 10.1	45.9 ± 8.5
	female	-	-	-	-	-

* *n* = 1.

## Data Availability

Detailed data sets of our measurement results can be found in Appendix A of this article.

## Appendix A

**Table A1:** Athletes’ age (mean ± standard deviation (=SD), years); athletes subdivided according to starting speed.

Performance Group (Starting Speed)	Sex		Speed Stage			
			1	2	3	4
1 (3.00/3.25 m/s)	male	-	-	-	-	-
	female	*n* = 13	15.9 ± 1.2	15.9 ± 1.2	15.9 ± 1.2	15.9 ± 1.2
2 (3.50/3.75 m/s)	male	*n* = 11	17.6 ± 1.0	17.6 ± 1.0	17.6 ± 1.0	17.6 ± 1.0
	female	*n* = 22	17.6 ± 2.4	17.6 ± 2.4	17.6 ± 2.4	17.6 ± 2.4
3 (4.00/4.25 m/s)	male	*n* = 33	17.4 ± 1.1	17.4 ± 1.1	17.4 ± 1.1	17.4 ± 1.1
	female	*n* = 2	17.0 ± 0.0	17.0 ± 0.0	17.0 ± 0.0	17.0 ± 0.0
4 (4.50/4.75 m/s)	male	*n* = 5	22.6 ± 5.1	22.6 ± 5.1	22.6 ± 5.1	22.6 ± 5.1
	female	-	-	-	-	-

**Table A2:** Athletes’ body mass (mean ± SD, kg); athletes subdivided according to starting speed.

Performance Group (Starting Speed)	Sex		Speed Stage			
			1	2	3	4
1 (3.00/3.25 m/s)	male	-	-	-	-	-
	female	*n* = 13	52.0 ± 5.7	52.0 ± 5.7	52.0 ± 5.7	52.0 ± 5.7
2 (3.50/3.75 m/s)	male	*n* = 11	70.3 ± 7.9	70.3 ± 7.9	70.3 ± 7.9	70.3 ± 7.9
	female	*n* = 22	52.2 ± 4.6	52.2 ± 4.6	52.2 ± 4.6	52.2 ± 4.6
3 (4.00/4.25 m/s)	male	*n* = 33	66.2 ± 6.1	66.2 ± 6.1	66.2 ± 6.1	66.2 ± 6.1
	female	*n* = 2	53.6 ± 13.6	53.6 ± 13.6	53.6 ± 13.6	53.6 ± 13.6
4 (4.50/4.75 m/s)	male	*n* = 5	65.3 ± 3.4	65.3 ± 3.4	65.3 ± 3.4	65.3 ± 3.4
	female	-	-	-	-	-

**Table A3:** Athletes’ body height (mean ± SD, cm); athletes subdivided according to starting speed.

Performance Group (Starting Speed)	Sex		Speed Stage			
			1	2	3	4
1 (3.00/3.25 m/s)	male	-	-	-	-	-
	female	*n* = 13	169.6 ± 6.2	169.6 ± 6.2	169.6 ± 6.2	169.6 ± 6.2
2 (3.50/3.75 m/s)	male	*n* = 11	183.6 ± 6.4	183.6 ± 6.4	183.6 ± 6.4	183.6 ± 6.4
	female	*n* = 22	169.5 ± 5.3	169.5 ± 5.3	169.5 ± 5.3	169.5 ± 5.3
3 (4.00/4.25 m/s)	male	*n* = 33	180.9 ± 5.1	180.9 ± 5.1	180.9 ± 5.1	180.9 ± 5.1
	female	*n* = 2	166.1 ± 0.2	166.1 ± 0.2	166.1 ± 0.2	166.1 ± 0.2
4 (4.50/4.75 m/s)	male	*n* = 5	184.2 ± 5.8	184.2 ± 5.8	184.2 ± 5.8	184.2 ± 5.8
	female	-	-	-	-	-

**Table A4:** Step frequency (mean ± SD, 1/s); athletes subdivided according to starting speed.

Performance Group (Starting Speed)	Sex		Speed Stage			
			1	2	3	4
1 (3.00/3.25 m/s)	male	-	-	-	-	-
	female	*n* = 13	2.82 ± 0.09	2.83 ± 0.11	2.85 ± 0.11	2.88 ± 0.12
2 (3.50/3.75 m/s)	male	*n* = 11	2.70 ± 0.09	2.71 ± 0.08	2.73 ± 0.09	2.75 ± 0.10
	female	*n* = 22	2.82 ± 0.13	2.85 ± 0.14	2.88 ± 0.14	2.91 ± 0.14
3 (4.00/4.25 m/s)	male	*n* = 33	2.73 ± 0.10	2.76 ± 1.10	2.80 ± 0.11	2.83 ± 0.12
	female	*n* = 2	2.95 ± 0.05	2.98 ± 0.06	3.02 ± 0.03	3.05 *
4 (4.50/4.75 m/s)	male	*n* = 5	2.77 ± 0.15	2.78 ± 0.17	2.81 ± 0.19	2.86 ± 0.19
	female	-	-	-	-	-

* *n* = 1 (as in all following tables, where indicated).

**Table A5:** Step frequency (mean ± SD, 1/min); athletes subdivided according to starting speed.

Performance Group (Starting Speed)	Sex		Speed Stage			
			1	2	3	4
1 (3.00/3.25 m/s)	male	-	-	-	-	-
	female	*n* = 13	169.2 ± 5.4	169.8 ± 6.6	171± 6.6	172.8 ± 7.2
2 (3.50/3.75 m/s)	male	*n* = 11	162 ± 5.4	162.6 ± 4.8	163.8 ± 5.4	165 ± 6
	female	*n* = 22	169.2 ± 7.8	171 ± 8.4	172.8 ± 8.4	174.6 ± 8.4
3 (4.00/4.25 m/s)	male	*n* = 33	163.8 ± 6	165.6 ± 6.6	168 ± 6.6	169.8 ± 7.2
	female	*n* = 2	177 ± 3	178.8 ± 3.6	181.2 ± 1.8	183 *
4 (4.50/4.75 m/s)	male	*n* = 5	166.2 ± 9	166.8 ± 10.2	168.6 ± 11.4	171.6 ± 11.4
	female	-	-	-	-	-

* *n* = 1.

**Table A6:** Ground contact time (mean ± SD, ms); athletes subdivided according to starting speed.

Performance Group (Starting Speed)	Sex		Speed Stage			
			1	2	3	4
1 (3.00/3.25 m/s)	male	-	-	-	-	-
	female	*n* = 11	195 ± 9	190 ± 9	184 ± 8	178 ± 8
2 (3.50/3.75 m/s)	male	*n* = 9	194 ± 15	188 ± 14	182 ± 14	177 ± 14
	female	*n* = 16	186 ± 14	180 ± 14	174 ± 14	169 ± 14
3 (4.00/4.25 m/s)	male	*n* = 31	182 ± 12	178 ± 12	173 ± 11	168 ± 11
	female	*n* = 2	176 ± 4	173 ± 5	167 ± 4	166 *
4 (4.50/4.75 m/s)	male	*n* = 2	176 ± 14	171 ± 14	167 ± 16	163 ± 17
	female	-	-	-	-	-

* *n* = 1.

**Table A7:** Time of flight (mean ± SD, ms); athletes subdivided according to starting speed.

Performance Group (Starting Speed)	Sex		Speed Stage			
			1	2	3	4
1 (3.00/3.25 m/s)	male	-	-	-	-	-
	female	*n* = 11	161 ± 10	166 ± 12	170 ± 11	173 ± 10
2 (3.50/3.75 m/s)	male	*n* = 9	177 ± 13	182 ± 13	185 ± 15	188 ± 17
	female	*n* = 16	168 ± 17	171 ± 18	171 ± 17	174 ± 18
3 (4.00/4.25 m/s)	male	*n* = 31	185 ± 11	186 ±11	186 ± 11	187 ± 11
	female	*n* = 2	162 ± 2	162 ± 2	163 ± 0.5	162 *
4 (4.50/4.75 m/s)	male	*n* = 2	201 ± 28	202 ± 29	203 ± 30	200 ± 30
	female	-	-	-	-	-

* *n* = 1.

**Table A8:** Amplitude of vertical oscillation (mean ± SD, cm) as measured at the sacrum; athletes subdivided according to starting speed.

Performance Group (Starting Speed)	Sex		Speed Stage			
			1	2	3	4
1 (3.00/3.25 m/s)	male	-	-	-	-	-
	female	*n* = 8	9.6 ± 0.9	9.6 ± 1.1	9.4 ± 1.3	9.4 ± 1.4
2 (3.50/3.75 m/s)	male	*n* = 11	11.0 ± 1.1	10.9 ± 0.9	10.9 ± 1.1	10.8 ± 1.1
	female	*n* = 21	9.9 ± 1.2	9.7 ± 1.1	9.5 ± 1.0	9.3 ± 1.0
3 (4.00/4.25 m/s)	male	*n* = 27	10.8 ± 1.1	10.5 ± 1.1	10.2 ± 1.0	9.9 ± 1.1
	female	*n* = 2	8.8 ± 0.5	8.7 ± 0.6	8.4 ± 0.5	4.2 ± 5.9
4 (4.50/4.75 m/s)	male	*n* = 5	10.5 ± 1.9	10.5 ± 2.0	10.1 ± 2.1	9.8 ± 1.8
	female	-	-	-	-	-

**Table A9:** Elevation of sacrum during flight (mean ± SD, cm); athletes subdivided according to starting speed.

Performance Group (Starting Speed)	Sex		Speed Stage			
			1	2	3	4
1 (3.00/3.25 m/s)	male	-	-	-	-	-
	female	*n* = 11	3.2 ± 0.4	3.4 ± 0.5	3.5 ± 0.5	3.7 ± 0.4
2 (3.50/3.75 m/s)	male	*n* = 9	3.9 ± 0.6	4.1 ± 0.6	4.2 ± 0.7	4.3 ± 0.8
	female	*n* = 16	3.5 ± 0.7	3.6 ± 0.8	3.6 ± 0.7	3.7 ± 0.8
3 (4.00/4.25 m/s)	male	*n* = 31	4.2 ± 0.5	4.2 ± 0.5	4.3 ± 0.5	4.3 ± 5.2
	female	*n* = 2	3.2 ± 0.1	3.2 ± 0.1	3.3 ± 0.0	3.2 *
4 (4.50/4.75 m/s)	male	*n* = 2	5.0 ± 1.4	5.1 ± 1.4	6.2	5.0 ± 1.5
	female	-	-	-	-	-

* *n* = 1.

**Table A10:** Step length (mean ± SD, cm); athletes subdivided according to starting speed.

Performance Group (Starting Speed)	Sex		Speed Stage			
			1	2	3	4
1 (3.00/3.25 m/s)	male	-	-	-	-	-
	female	*n* = 13	115 ± 5	123 ± 6	131 ± 6	138 ± 7
2 (3.50/3.75 m/s)	male	*n* = 11	139 ± 7	147 ± 6	155 ± 6	163 ± 7
	female	*n* = 22	128 ± 7	134 ± 7	142 ± 8	149 ± 8
3 (4.00/4.25 m/s)	male	*n* = 33	152 ± 6	159 ± 6	166 ± 6	173 ± 7
	female	*n* = 2	135 ± 2	142 ± 3	149 ± 2	156 *
4 (4.50/4.75 m/s)	male	*n* = 5	166 ± 7	175 ± 8	182 ± 9	188 ± 10
	female	-	-	-	-	-

* *n* = 1.

**Table A11:** Oxygen intake (mean ± SD, ml·kg^−1^·min^−1^); athletes subdivided according to starting speed.

Performance Group (Starting Speed)	Sex		Speed Stage			
			1	2	3	4
1 (3.00/3.25 m/s)	male	-	-	-	-	-
	female	*n* = 13	39.2 ± 2.6	42.8 ± 2.6	45.5 ± 2.5	48.2 ± 1.1
2 (3.50/3.75 m/s)	male	*n* = 11	47.4 ± 3.6	50.4 ± 3.6	53.2 ± 3.7	55.8 ± 3.4
	female	*n* = 22	43.1 ± 2.7	46.8 ± 2.8	49.8 ± 2.9	52.8 ± 1.2
3 (4.00/4.25 m/s)	male	*n* = 33	50.0 ± 3.2	53.5 ± 3.4	56.7 ± 3.2	60.3 ± 5.7
	female	*n* = 2	46.8 ± 0.5	50.8 ± 0.1	55.0 ± 0.3	57.4 *
4 (4.50/4.75 m/s)	male	*n* = 5	51.5 ± 8.3 *	55.4 ± 8.5 *	58.3 ± 6.6	63.6 ± 3.9
	female	-	-	-	-	-

* *n* = 1.

**Table A12:** Blood lactate accumulation (mean ± SD, mmol L^−1^); athletes subdivided according to starting speed.

Performance Group (Starting Speed)	Sex		Speed Stage			
			1	2	3	4
1 (3.00/3.25 m/s)	male	-	-	-	-	-
	female	*n* = 13	1.32 ± 0.36	1.70 ± 0.63	2.62 ± 1.04	4.22 ± 2.04
2 (3.50/3.75 m/s)	male	*n* = 11	1.75 ± 0.48	2.34 ± 0.65	3.53 ± 1.03	5.50 ± 1.61
	female	*n* = 22	1.22 ± 0.38	1.68 ± 0.44	2.65 ± 0.87	4.48 ± 1.51
3 (4.00/4.25 m/s)	male	*n* = 33	1.49 ± 0.52	1.90 ± 0.59	2.83 ± 0.80	4.54 ± 1.25
	female	*n* = 2	1.34 ± 0.21	2.05 ± 0.44	3.64 ± 1.04	4.86 *
4 (4.50/4.75 m/s)	male	*n* = 5	1.17 ± 0.42	1.55 ± 0.54	2.40 ± 0.78	4.38 ± 1.38
	female	-	-	-	-	-

* *n* = 1.

**Table A13:** Aerobic contribution to C_r_ (mean ± SD, J·kg^−1^·m^−1^); athletes subdivided according to starting speed.

Performance Group (Starting Speed)	Sex		Speed Stage			
			1	2	3	4
1 (3.00/3.25 m/s)	male	-	-	-	-	-
	female	*n* = 13	3.76 ± 0.24	3.86 ± 0.24	3.85 ± 0.21	3.87 ± 0.17
2 (3.50/3.75 m/s)	male	*n* = 11	4.01 ± 0.29	4.05 ± 0.30	4.06 ± 0.30	4.03 ± 0.25
	female	*n* = 22	3.75 ± 0.23	3.84 ± 0.22	3.90 ± 0.22	3.93 ± 0.26
3 (4.00/4.25 m/s)	male	*n* = 33	3.83 ± 0.23	3.90 ± 0.24	3.95 ± 0.22	4.03 ± 0.37
	female	*n* = 2	3.69 ± 0.04	3.82 ± 0.02	3.95 ± 0.03	3.93 *
4 (4.50/4.75 m/s)	male	*n* = 5	3.51 ± 0.73	3.62 ± 0.72	3.70 ± 0.51	3.90 ± 0.27
	female	-	-	-	-	-

* *n* = 1.

**Table A14:** Anaerobic contribution to C_r_ (mean ± SD, J·kg^−1^·m^−1^); athletes subdivided according to starting speed.

Performance Group (Starting Speed)	Sex		Speed Stage			
			1	2	3	4
1 (3.00/3.25 m/s)	male	-	-	-	-	-
	female	*n* = 13	0.037 ± 0.06	0.013 ± 0.01	0.029 ± 0.02	0.051 ± 0.03
2 (3.50/3.75 m/s)	male	*n* = 11	0.018 ± 0.01	0.018 ± 0.01	0.037 ± 0.01	0.063 ± 0.03
	female	*n* = 22	0.010 ± 0.01	0.014 ± 0.01	0.031 ± 0.01	0.058 ± 0.02
3 (4.00/4.25 m/s)	male	*n* = 33	0.015 ± 0.01	0.011 ± 0.00	0.026 ± 0.01	0.048 ± 0.02
	female	*n* = 2	0.010 ± 0.00	0.018 ± 0.00	0.040 ± 0.00	0.06 *
4 (4.50/4.75 m/s)	male	*n* = 5	0.014 ± 0.01	0.010 ± 0.00	0.021 ± 0.01	0.049 ± 0.02
	female	-	-	-	-	-

* *n* = 1.

**Table A15:** Total C_r_ (mean ± SD, J·kg^−1^·m^−1^); athletes subdivided according to starting speed.

Performance Group (Starting Speed)	Sex		Speed Stage			
			1	2	3	4
1 (3.00/3.25 m/s)	male	-	-	-	-	-
	female	*n* = 13	3.77 ± 0.25	3.87 ± 0.25	3.88 ± 0.22	3.92 ± 0.17
2 (3.50/3.75 m/s)	male	*n* = 11	4.03 ± 0.29	4.06 ± 0.30	4.09 ± 0.30	4.09 ± 0.27
	female	*n* = 22	3.76 ± 0.23	3.86 ± 0.22	3.92 ± 0.22	3.99 ± 2.64
3 (4.00/4.25 m/s)	male	*n* = 33	3.85 ± 0.24	3.92 ± 0.24	3.98 ± 0.20	4.06 ± 0.37
	female	*n* = 2	3.70 ± 0.04	3.84 ± 0.02	3.99 ± 0.04	3.99 *
4 (4.50/4.75 m/s)	male	*n* = 5	3.51 ± 0.72	3.63 ± 0.72	3.72 ± 0.52	3.95 ± 0.29
	female	-	-	-	-	-

* *n* = 1.
